# The Power of Modeling in Emergency Preparedness for COVID-19: A Moonshot Moment for Hospitals

**DOI:** 10.1017/dmp.2021.51

**Published:** 2021-02-16

**Authors:** Kyan C. Safavi, Ann L. Prestipino, Ana Cecilia Zenteno Langle, Martin Copenhaver, Michael Hu, Bethany Daily, Allison Koehler, Paul D. Biddinger, Peter F. Dunn

**Affiliations:** 1 Healthcare Systems Engineering, Massachusetts General Hospital, Boston, MA, USA; 2 Department of Anesthesia, Critical Care and Pain Medicine, Massachusetts General Hospital, Boston, MA, USA; 3 Department of Perioperative Services, Massachusetts General Hospital, Boston, MA, USA; 4 Hospital Administration, Massachusetts General Hospital, Boston, MA, USA; 5 Center for Disaster Medicine, Massachusetts General Hospital, Boston, MA, USA

**Keywords:** hospital bed capacity, health care facilities, manpower and services, surge capacity, decision making, organizational, delivery of health care

## Abstract

Before coronavirus disease 2019 (COVID-19), few hospitals had fully tested emergency surge plans. Uncertainty in the timing and degree of surge complicates planning efforts, putting hospitals at risk of being overwhelmed. Many lack access to hospital-specific, data-driven projections of future patient demand to guide operational planning. Our hospital experienced one of the largest surges in New England. We developed statistical models to project hospitalizations during the first wave of the pandemic. We describe how we used these models to meet key planning objectives. To build the models successfully, we emphasize the criticality of having a team that combines data scientists with frontline operational and clinical leadership. While modeling was a cornerstone of our response, models currently available to most hospitals are built outside of their institution and are difficult to translate to their environment for operational planning. Creating data-driven, hospital-specific, and operationally relevant surge targets and activation triggers should be a major objective of all health systems.

Hospitals have spent years creating emergency plans to surge inpatient capacity, but before coronavirus disease 2019 (COVID-19), few such plans had been fully tested.^1-3^ When determining how best to activate plans for pandemics and other disasters, hospitals must estimate how much clinical demand they may experience and calibrate their actions to match the need. In this environment of uncertainty, however, many lack access to data-driven projections of future patient needs with sufficient detail to guide operational planning. When planning targets are not driven by objective data and analyzed with appropriate statistical and modeling tools, effective planning becomes challenging.^4^

Our hospital, which had one of the highest COVID-19 surges in the Northeast during the first wave, developed statistical models to project patient hospitalizations for surge planning. These targets were used to mobilize and coordinate resources in real time, identify and address gaps, and trigger phases of our surge plan with appropriate lead time to open new units safely.

Years before COVID-19, our hospital established a division, Healthcare Systems Engineering, with expertise in data modeling to help guide leadership in solving operational challenges. For all projects, data scientists and leaders in the hospital’s clinical and operational realms worked together as a single team. Examples of projects include the optimization of hospital bed capacity to reduce emergency department (ED) waiting times and the realignment of operating room schedules to reduce downstream bottlenecks. The data models helped leaders gain insights into their environment and provide solution options and an estimation of the impact of each on the hospital and its patients.^5-7^

For the COVID-19 response, the hospital activated its emergency preparedness plan including the hospital incident command system (HICS), which coordinated all planning efforts. The HICS incorporated Healthcare Systems Engineering with representatives from each surge planning section, including bed capacity, staffing, equipment, and supply chain. At daily HICS meetings, team members vetted what data were operationally useful to guide model design. Model results were shared with frontline leaders, and their feedback helped refine the modeling approach. Finalized results were communicated broadly, ensuring that an institution-wide understanding of the analysis was established across all aspects of the planning effort.

Modeling played a critical role in 2 essential objectives of disaster planning for our hospital. First, given the significant uncertainty at the beginning of the pandemic and the substantial lead-time required for action, we developed data-driven planning targets for a worst-case scenario using a mechanistic model that estimated peak occupancy many weeks in advance.^8^ These estimates had substantial variation, but gave our hospital a framework to develop detailed surge plans and to evaluate the adequacy of these plans for general care and intensive care unit (ICU) beds, ventilators, and personal protective equipment (PPE) against the maximum projected needs.

The basis of this approach was to predict peak occupancy scenarios from mild to severe. Assumptions regarding community infection rate, symptomatic rate, hospitalization ratios, critical care use, and length of stay (LOS) were applied to the hospital’s estimated catchment geography. Given the uncertainty of these assumptions during this early period of the pandemic, we ran dozens of sensitivity analyses on each of these parameters. The initial mild versus severe scenarios estimated an adult general care peak occupancy of 84 and 362 and an ICU peak occupancy of 75 and 301, respectively.

We also used this approach to support our broader hospital system. Using the severe scenario as a target, hospitals within our system submitted resource mobilization plans to HICS leaders, who identified gaps between surge capacity and their modeled peak demand. HICS leaders applied best practices across the system so that sites with inadequate planning could better optimize their patient care capacity. In addition, special resource sharing mechanisms were established to support hospitals who were especially vulnerable based on the models. These included establishing a centralized ventilator supply and distribution mechanism and an oversight group to direct daily inpatient transfers from hospitals with severely strained resources to those with available capacity.

As COVID-19 hospitalizations increased during the early weeks of the pandemic, the team began to accumulate data on actual occupancy within our hospital. We then fit the model’s curve to our actual occupancy data. We used this fitted curve to update our initial assumptions of model inputs to be more precise. The updated curve allowed us to track our hospital’s trajectory relative to the mild and severe benchmarks that were originally created. This helped improve our hospital and system leadership’s awareness of our expected trajectory, which tracked within the mild and extreme benchmarks peaking at 237 general care patients and 177 ICU patients.

The second objective was to forecast general care and ICU occupancy in 14-d increments, which provided enough lead-time to make operational decisions about whether and when to open surge units. The forecasts guided activation of each surge phase by matching bed supply to upcoming demand. Operationalizing surge units before an urgent need developed meant that patients could gradually flow into them, which helped staff in these nontraditional spaces safely learn how to work in those areas and gain confidence. It also ensured that surge spaces were not activated prematurely with prolonged periods of under-use.

As we gathered more data during the pandemic period, the modeling continuously evolved. Leveraging data from the hundreds and eventually thousands of COVID-19 patients who had been discharged from our system, we transitioned to a simulation model. This model could estimate the remaining hospital course of currently admitted and future patients by randomly selecting among prior hospitalizations. We further refined the model by accounting for differences in the hospital course of subgroups of COVID-19 patients with distinct care pathways. These included index admissions versus readmissions, ED admissions versus inpatient transfers, and patients whose primary reason for admission was driven by COVID-19 versus by other disease processes (eg, cerebral hemorrhage). We also included non–COVID-19 patients into the model given their growing contribution to the hospital occupancy as ED and elective procedural volume returned. Model performance during a 2-mo period of the first wave of the pandemic had an average absolute percentage error of 2.8% for total occupancy.

Modeled projections and related surge plans were communicated with the entire hospital workforce in weekly virtual townhall meetings. This transparency reduced uncertainty and, as the workforce followed the data over time, built confidence in the hospital’s pandemic response. One challenge that we encountered was communicating the exact numbers predicted by the models while ensuring that our audience simultaneously understood the uncertainty in these predictions and did not rigidly plan to these parameters. Our models ran 100 iterations for reach prediction, and we presented the range of results to emphasize this uncertainty. In particular for operational planning leadership, we reiterated the importance of using the model output to be directionally sound in our planning approach and timing, but to remain flexible to pivot as needed based on our actual lived experience.

The data were also used to communicate anticipated resource needs with the state and federal government, including PPE and post-acute space. For example, the modeled peak occupancy for the health system was used to estimate post-acute care occupancy, which helped evaluate the potential role of a government-funded post-acute care surge facility at the Boston Convention Center.

While our internal modeling was a cornerstone of our response, the models currently available to many hospitals are often built outside of their institution. These models typically generate predictions at a regional level, making them difficult to translate into operational plans for individual hospitals or systems.^9^ The city and state models typically use data at a population level and make hospital occupancy projections based on community case positivity. These models, while potentially useful for guiding community-based interventions, are less useful for hospitals. Our models emphasize hospital admissions rather than cases in the community as a lead indictor. We found that hospital admissions were less affected by variables such as testing availability and turnaround time, among others, which could impact community case rates. Furthermore, admissions were much more closely tied to hospital occupancy and, therefore, useful to hospital operations.

In addition, the data scientists and epidemiologists who create state-wide models are not always familiar with hospital operational concerns and must often work independently from the clinical leaders who orchestrate the surge, making their projections less responsive to the on-the-ground needs of those caring for patients. Both sides must come together to builds models that can meaningfully guide hospital surge efforts.

Creating the capability to develop data-driven, hospital-specific, and operationally relevant surge targets and activation triggers should be a major objective of health systems across the country. While building such teams may be out of reach for some, this capability can be shared within and across systems, if the focus on hospital-specific operations is preserved. The data needed in the models are standard and available for every hospital, including COVID admission rates, ICU use, LOS, and catchment population. The data used within our models were updated every 24 h in an institutional administrative database linked to our electronic medical record. Most hospitals throughout the United States now have electronic medical records and should have the same data access and cadence of data updates.

A future direction for consideration is to provide access across hospital systems to data scientists who have the expertise to build these models, although it is essential that they partner with operational and disaster leaders within the individual sites. To help increase the dissemination of knowledge to these groups, hospitals such as ours that have developed such models and assessed their effectiveness must make their underlying methodologies and technical specifications available by means of open access to others.

Perhaps more daunting than the modeling is the idea of meaningful cross-collaboration in a health-care disaster response. We must rid ourselves of the fallacy that hospital systems are completely independent, as their fates are linked now more than ever before. If 1 hospital becomes overwhelmed due to insufficient planning, others will be forced to compensate. We must rethink our expectations about state and region-wide collaboration. Boston, for example, has a long history of inter-hospital collaboration around disaster planning, and during the first surge, 6 health systems serving the entire population of Eastern Massachusetts worked together to ensure that no individual system was overwhelmed.^10^ The tools and insights generated by modeling efforts that link data scientists and operational leaders can be spread through these kinds of collaborations, which we must foster throughout the country. No hospital or patient should be left behind.
